# Beyond Innovation: The Burdens and Harms of Maintenance in Deep Brain Stimulation

**DOI:** 10.1007/s11948-026-00628-6

**Published:** 2026-09-11

**Authors:** Marilena Pateraki, Frederic Gilbert

**Affiliations:** 1 Department of History and Philosophy of Science, National Kapodistrian University of Athens, Athens, Greece; 2 School of Humanities, Philosophy and Gender Studies, University of Tasmania, Hobart, Australia

**Keywords:** Incorporation, Deep brain stimulation, Ethics, Harms, Maintenance, Obsolescence

## Abstract

In this contribution, we explore iatrogenic harms associated with the maintenance of deep brain stimulation (DBS) systems. By conceptualizing patients with neural implants as infrastructures, we examine them as ongoing sites of maintenance that require continual care and adjustments. This analysis reveals the tradeoffs inherent in implantable neurotechnologies. Using first-hand accounts collected during a qualitative study of individuals with Parkinson’s disease treated with DBS, we subsequently develop a typology of harms associated with maintenance, highlighting the ethical issues arising from the integration of technology in the body. Importantly, by focusing on older DBS devices within a healthcare system constrained by financial resources, we address a gap in the literature, as current debates tend to prioritize innovation while neglecting the difficulties of maintaining older implanted systems. Our findings show how inadequate or faulty maintenance compromises the successful incorporation and integration of technological components within the body, underscoring the ethical significance of maintenance in the context of DBS. Finally, we emphasize the inherent limitations of implanted technologies in managing chronic illness and the enduring importance of caregivers. This study calls for a more comprehensive examination of the ethical implications of the maintenance in relation to neural implants.

## Introduction

Deep brain stimulation (DBS) is a subcutaneous technological system that often goes unnoticed by both users and observers, rendering it an essentially invisible technology. Despite its unobtrusive appearance, DBS exerts a pervasive and significant influence on patients’ lives because of its deeply invasive integration within the neural system. It entails a neurosurgical procedure involving the implantation of electrodes in specific brain regions to alleviate symptoms associated with conditions such as Parkinson’s disease (PD). These electrodes deliver electrical impulses powered by an implanted pulse generator (IPG) in the chest, which modulate neural activity. DBS is an approved, safe, and effective treatment for several neurological conditions, including PD, dystonia, and essential tremor. It is considered an experimental intervention for certain neuropsychiatric disorders, such as major depressive disorder, anorexia nervosa, and addiction. The number of patients receiving implants continues to rise, driven by several factors. First, the range of medical conditions targeted by implants has expanded, broadening indications for their use (Lozano et al., 2019; Krauss et al., 2021). Second, improvements in healthcare infrastructure and technology have increased accessibility to DBS treatment (Sarica et al., 2023; Gilbert et al., 2023a). Third, longitudinal studies suggest that DBS may improve life expectancy among recipients (Henriksen et al., 2016; Gilbert & Lancelot, 2021; Lancelot, 2019, 2022; Merola et al., 2014; Ngoga et al., 2014; Weaver et al., 2017). An estimated 250,000 individuals worldwide use DBS, with further growth expected due to population aging (Lee et al., 2019). However, as this number increases, questions arise regarding how DBS recipients coexist with their invisible neural devices. Rapid advances in neuroscience and clinical technology mean that today’s cutting-edge devices may soon become outdated and fall into desuetude, leaving older neurotechnologies vulnerable to obsolescence or increased maintenance demands (Okun et al., 2024).

A substantial portion of the neuroethical literature focuses on DBS’s putative effects on alterations in patients’ personality, identity, agency, autonomy, authenticity and self (PIAAAS) (Gilbert et al., 2017, 2021a, 2021b; Bluhm & Cabrera, 2021; Furlanis & Gilbert, 2023; Pugh et al., 2021; Mosley et al., 2019; Snoek et al., 2021; Erler, 2021; Zuk & Lázaro-Muñoz, 2021; Kubu et al., 2021; Thomson et al., 2021). While ethically significant, debates centered on PIAAAS often focus narrowly on one avenue of inquiry, overlooking the materialities and caring practices involved in neurostimulation (Pateraki, 2022, 2023). These practices are crucial because, in implants, the technological infrastructure becomes an integral part of patients’ bodies, requiring ongoing maintenance (Gilbert et al., 2023b; Bublitz & Gilbert, 2023; Gilbert et al., 2019; Forlano, 2017a, 2017b; Pateraki, 2022, 2022). Consequently, living with an implant entails ongoing maintenance; a perspective that may reframe ethical issues in DBS. Although literature on the upkeep of innovative implantable devices is growing (e.g. Dasgupta et al., 2024; Lázaro-Muñoz et al., 2022; Fins et al., 2024), maintenance of older DBS systems has received little attention. This is significant, as many patients continue to rely on such devices, underscoring the importance of examining the ethical challenges they pose. By addressing this gap and drawing on first-hand narratives, our study provides novel insights into the often-overlooked ethical dimensions of maintaining older DBS systems for PD within resource-limited healthcare settings.

This article focuses on the ethics of DBS maintenance and the potential burdens and harms it entails. By conceptualizing bodies with neural implants as sites of maintenance, we aim to shed light on the epistemic value of the harms through the lens of maintenance. This study foregrounds the experiences of patients living with older DBS systems, filling a gap in the literature that often privileges innovation at the expense of reflecting on the ethical challenges of maintaining legacy technologies. We will build our work upon narratives collected from DBS users, providing novel evidence for the ways harms and maintenance often intertwine. More specifically, we examine how the need for, or lack of, maintenance shapes the integration and incorporation of technological components within the body. Inadequate maintenance can trigger recurrent and rebound symptoms, along with psychological effects, potentially imposing unforeseen burdens on both patients and caregivers (Gilbert et al., 2024).

Analytically, the first section explains what maintenance entails in the context of DBS. The second section examines patients’ narratives to explore how iatrogenic harms and burdens interfere with maintenance. This part consists of four subsections. First, we present our research methodology. Second, we analyze the trade-offs of DBS, focusing on maintenance practices and illustrating how the merging of human and machine produces both benefits and harms for patients. Third, we identify four types of harms associated with DBS maintenance. Finally, we discuss the challenges and constraints that arise in specific cases. The third section synthesizes these findings, introduces a typology of harms related to DBS maintenance, and highlights the critical need for their prompt and effective management.

### Maintenance: A Necessary Process in DBS Incorporation

In this section, we examine what maintenance entails in the context of DBS. We argue that incorporation is a fundamental, though often overlooked, dimension of maintenance in under-the-skin technologies, albeit not the only one. To begin, a few clarifications are necessary. It is crucial to recognize that any implanted device exists within a “temporal trajectory,” that is, the “life cycle” or “lifespan” of technology, one shaped by durability and obsolescence. The term durability in neurotechnology refers to the length of time a device or system remains functional and effective, requiring minimal maintenance or repair over its useful life. This concept applies not only to individual devices but also to their iterations (e.g., versions 1.0, 2.0, and beyond) and categories (e.g., unipolar versus multipolar DBS electrodes) of therapeutic tools (Okun et al., 2024). As newer and more sophisticated types of implantable devices become commercially available, older models of neurotechnology may face obsolescence or increased maintenance challenges (Ibid).

According to the philosopher of technology Mark Thomas Young, maintenance is “an activity through which we provide stability to something over time.” (Young, 2020, p. 356) This activity is particularly relevant for implanted devices, where maintenance requires ongoing adjustments to ensure proper functioning and support patient well-being. Maintenance raises concerns about technological obsolescence and user experience, but also neurotechnological fatigue: where users grow weary of interacting with or relying on an obsolete device, even if it remains functionally intact. This fatigue may stem from the constant impossibility to update, or from the psychological burden of being tethered to a technology that no longer feels adequate or aligned with evolving standards. In turn, it may lead to abandonment and raise ethical questions about sustainability, user autonomy, and long-term viability of neurotechnological interventions. These issues also prompt reflection on how such devices are integrated into long-term care, and how healthcare systems allocate resources to support technologies that remain vital to patients despite being surpassed by newer innovations. Addressing these issues is essential to grasp the ethical stakes of maintaining neurotechnologies.

In this rapidly evolving landscape of implantable neurotechnologies, we recognize that the individuals receiving these implants are more than just passive recipients of a system (Pateraki, 2026). They are, in fact, active components within the neural technology ecosystem (Ibid). Implants are continuously and inextricably intertwining with the body (Oudshoorn, 2016), while caring for these bodies is relational and tailored, attentive to symptom fluctuations and the progression of the disease (Mol, 2008p. 18, 23). Maintenance of the embedded technology becomes a core aspect of chronic care (Forlano, 2017a; Pateraki, 2022, 2023), blurring the boundaries between caring for the person and maintaining the device (Pateraki, 2022, 2023). Despite its importance, maintenance has received limited attention in research on disability-related technologies, with some notable exceptions (Oudshoorn, 2015; Shew, 2023; Forlano, 2017a, b; Earle, 2019). This framing allows us, first, to consider bodies with neural implants as infrastructures, sites where the ongoing labor of sustaining technological function occurs, and second, to view DBS incorporation as a process extending beyond mere durability.

Yet, the question of what “maintenance” entails in the context of DBS for PD remains open. As we argue, a crucial aspect of maintenance is incorporation. Generally, incorporation refers to the process through which an artifact becomes a transparent part of bodily functioning: it is no longer perceived as an external object but is integrated into bodily habituations (Ihde, 1990, p. 7; Merleau-Ponty, 2012). When successful, this process creates a human-technology relation in which artifacts are functionally and experientially integrated into the body (Merleau-Ponty, 2012). In this state of transparency, as Ihde (1990, p. 75) notes, the artifact is absorbed into bodily routines, much like eyeglasses, which are often not experienced as external objects by their wearer. Incorporation in this sense denotes that technology becomes a seamless part of bodily functioning, operating without drawing conscious attention.

In DBS, incorporation entails a successful coupling between patient and device, achieved and sustained through continuous effort and adjustment (Pateraki, 2022, 2023, 2026). In this case, incorporation extends from the surgical placement of the device and initial programming to ongoing calibration, IPG replacement, medication adjustment, the avoidance of harmful environments, and other practices, each involving continuous adjustment and attention to ensure the device functions according to the patient’s needs (Ibid). Technology does not become transparent or fully integrated on its own; achieving optimal functionality for the patient depends on ongoing work to adapt the device to the fluctuations of the symptoms. All of the above must occur for incorporation to take place, and, additionally, for the device to be accepted by the persons as part of themselves, to some degree, sometimes experienced as a symbiotic component (Gilbert et al., 2023b; Bublitz & Gilbert, 2023; Gilbert et al., 2019).

These are non-urgent, ongoing maintenance activities that support incorporation and are intended to adapt the device to the patient’s evolving symptoms (Pateraki, 2022, 2023). These activities help preserve the patient’s well-being and quality of life over time (Furlanis & Gilbert, 2023). Even when there is no immediate danger, these practices constitute maintenance because they sustain the functionality, stability, and reliability of the DBS system over time, supporting the patient’s everyday functioning and quality of life. In this sense, maintenance is not merely reactive; it is a proactive, continuous process of aligning technology with bodily realities, reflecting the intricate interdependence between patient and device.

Furthermore, in the context of DBS, maintenance involves a range of activities that ensure the device continues to function safely, effectively, and in alignment with the patient’s needs. These are urgent interventions aiming at preventing serious harms, such as implant leakage, hemorrhage, or migration, which demand immediate clinical attention (Maslen et al., 2018; Coroners Court of Queensland, 2017; State Coroner’s Court of New South Wales, 2014; Gilbert, 2013). These activities are considered maintenance because they preserve the device’s integrity and prevent life-threatening failures, highlighting that care extends beyond the patient to the technological system itself.

Ultimately, maintenance lies at the core of DBS incorporation. It is through the continuous and often complex work of sustaining device functionality, ranging from urgent interventions to prevent serious harms to routine practices such as calibration and IPG replacements, that long-term alignment with patients’ evolving needs is achieved. Maintenance not only ensures the technical stability and reliability of the DBS system, but also enables the device to become transparent in bodily functioning, supporting patients’ autonomy, well-being, and quality of life. By foregrounding maintenance, we highlight that the successful incorporation of DBS is not a one-time event but an ongoing relational process, reflecting the intricate interdependence between patient and technology. Recognizing this centrality of maintenance allows us to appreciate the ethical stakes involved in caring for both the person and the technological system over time.

In the case of DBS users, stability is the result of a continuous process of maintenance, leading to incorporation. In showing the processes of maintenance and the harms related to them, we argue that maintenance stands to the heart of incorporation.

### Patients’ Narratives: Evidence of How Harm Intertwines with Maintenance

Examining the experiences of DBS patients with older devices in healthcare systems with limited resources brings maintenance issues, rarely addressed in the literature, into sharp focus. In the following subsections, we present findings from a two-and-a-half-year field study conducted in Greece. Before doing so, we first outline our research methodology.

#### Research Methodology

The data were collected between 2016 and 2018 as part of the doctoral requirements of the first author’s PhD research project. She conducted the data collection and was responsible for the subsequent processing and analysis. The study draws on forty-five interviews: nineteen with PD patients who had undergone DBS in Greece; thirteen with caregivers (relatives and professionals); five with PD activists without DBS; and eight with health professionals. Patients were recruited through a combination of clinical contacts and personal networks. Two neurologists with expertise in DBS, working in major public hospitals in Attica, facilitated access to some participants, while additional contacts with four DBS patients were established through existing personal networks. The sample included patients with diverse symptom profiles and different stages of PD, capturing heterogeneous experiences of rejecting, living with, and adapting to DBS. In terms of gender distribution, the ratio of men to women was almost 1:1 among patients and caregivers. Among health professionals, however, the ratio was 4:1, reflecting the gender imbalance across the clinical specialties involved in DBS.

Semi-structured, in-depth interviews were conducted in Greek and lasted between thirty minutes and two hours, with an average duration of one hour and twenty-four minutes and a median length of one hour and five minutes. The interviews were recorded and transcribed verbatim, and the discursive data were complemented with observational notes. Data were collected and analysed using a Constructivist Grounded Theory approach (Charmaz, 2006), with coding performed concurrently with data collection. Using ATLAS.ti, data were iteratively coded and compared, while analytical memos were written throughout the process, leading to the development and refinement of conceptual categories grounded in participants’ experiences. Maintenance emerged as one of the key thematic categories through this process. The interview vignettes were organized around the analytic categories developed through the iterative process of coding and comparison. Interviews continued until thematic saturation was achieved, allowing for a thorough and nuanced exploration of participants’ experiences.

Consistent with Constructivist Grounded Theory, the researcher’s positionality was acknowledged throughout the knowledge production process. Reflexivity was maintained through reflexive journaling, memo writing during the coding process, continuous comparison of emerging interpretations, and critical discussions with her supervisor and colleagues to examine analytic decisions, challenge assumptions, and enhance the credibility of the analysis. This research was conducted before the EU General Data Protection Regulation (GDPR) was implemented in Greece through Law 4624/2019. Nevertheless, it adhered to strict ethical guidelines in accordance with the standards of the GDPR. All data were anonymized, and interviewees are referred to by pseudonyms to ensure privacy.

Patients’ narratives illuminate how maintenance issues directly affect the lives and well-being of DBS patients. The following sections present analytically selected vignettes that illustrate how maintenance is experienced, negotiated, and interpreted by patients and caregivers in everyday life. The vignettes were selected not only for their empirical richness but also for their capacity to address the theoretical concerns of the article. Rather than functioning as standalone illustrations, they serve as analytical devices connecting participants’ situated experiences with broader questions concerning the ongoing work required to sustain DBS as an implanted technology. Our findings reveal the trade-offs inherent in DBS use: while generally beneficial, DBS entails not only benefits but also harms for the patients, as well as varying degrees of burden for their caregivers.

#### “Brain Reparation with a Technology That Needs Repair?”: The Trade-Offs of Under-The-Skin Technologies

Takis, a 68-year-old man with PD and an activist, despite meeting the criteria for DBS, refused to undergo the procedure. He explained his decision during an interview at a PD patients’ association office as follows:

I wouldn’t like to have a gadget in my head, in my own brain! I don’t want that! (…) I don’t want my flesh to mingle with metal. To mingle with junk! (…) Let’s say you do perform the operation, who says that the gadgets won’t fail after a while? Are we sure they’re going to work? My own cell phone often does funny things. Listen, Miss, I’m sick and I always have to change stuff, with the doctors, medications, physiotherapy. And, assuming that [DBS] will work, of which I doubt … Will I always have to fix my gadgets too? I’m afraid! I do not have time for such things, I cannot be bothered anymore! Is such a future worthwhile? Brain reparation with a technology that needs repair too? I don’t trust neither technology nor our healthcare system! (…) In the Greek healthcare system, you don’t know if it will be there when you need it, if it will provide timely support for this technology, or if it can manage all the complications that might arise.

(Interview with Takis)

Takis’ testimony reveals an array of issues around DBS, explicitly pointing to maintenance concerns. Articulating the core elements of what Steven Jackson calls ‘broken world thinking’ (Jackson, 2014), he demonstrates a keen awareness of the fragility of the material world and, in this case, of DBS. Takis wants to avoid an under-the-skin technology, fearing potential harm due both to the fragility of the device and the lack of its maintenance, and expresses distrust toward the Greek healthcare system. His argument rests on two distinct elements: first, an emotional one, a visceral rejection of DBS as “the mingling of flesh and metal,” expressing a form of ontological fear; and second, a rational one, grounded in perceived technological fragility and inadequate healthcare infrastructure.

As a longtime PD patient, Takis is conscious of the web of dependencies surrounding chronic illness, including “doctors, medications, physiotherapy,” an array of actors and actants involved in the mechanism of his chronic care. Thus, he views DBS as adding another layer of complexity in his life. Already enmeshed in complex dependencies, he believes DBS exacerbates them, increasing “the complexity of the socio-technical system and its vulnerability to breakdown.” (Forlano, 2017a, p. 4; Pateraki, 2022) Furthermore, Takis’s concerns resonate with broader discussions about under-the-skin technologies, which may indeed enhance patients’ everyday lives but can also introduce new forms of vulnerability. Nelly Oudshoorn (2016), following Mark Coeckelberg (2013), has described this as “cyborg vulnerabilities” for patients with pacemakers and defibrillators. Sara Goering, Anna Wexler, and Eran Klein (2021) extend this idea by conceptualizing it as a process of “trading vulnerabilities,” highlighting risks and benefits inherent in living with these devices. From this perspective, DBS patients shift from bodily limitations associated with PD to technological and medical dependency, highlighting the dynamic and relational nature of vulnerability in chronic illness (Ibid).

As Takis explains, “In the Greek healthcare system, you don’t know if it will be there when you need it, if it will provide timely support for this technology, or if it can manage all the complications that might arise.” This highlights that maintenance and reliability are central to his evaluation of DBS, and reflects his broader concern that adding a technological layer could increase uncertainty, dependency, and the burden of managing his own treatment. He believes that inadequate maintenance would harm him further and is convinced that such potential harms would outweigh the possible benefits of the technology. To him, the prospect of entrapment within technology and the associated harms is completely intolerable.

Takis thus considers DBS only as a source of potential harms; nevertheless, his opinion despite being informed, remains speculative. In contrast, Antonia, a 56-year-old woman living with DBS, has experienced both its beneficial and harmful effects and understands the trade-offs involved (Ihde, 2008). She was diagnosed at 39 and underwent DBS nine years later. Now, after eight years, she knows well what living with the technology means:

Well, it takes a constant effort to be well. Initially, my access [to DBS] was delayed, and [afterwards] the implantation and its sequels were very painful. Furthermore, it [DBS] requires calibration, which adapts the device to my body, changes in medication, battery replacement [IPG], and I have to be careful as to where I am in order to avoid accidents. In other words, living with DBS is not so simple. Besides that, sometimes I’m well, I can do many things that I want and some other times I feel bad and that affects me

(Interview with Antonia)

Takis and Antonia both understand chronic illness and DBS incorporation as ongoing processes; however, while Takis sees DBS as fragile and potentially harmful, Antonia accepts DBS as part of herself. Antonia is acutely aware of the trade-offs involved in a technology that has become part of her body. Because it operates beneath the skin, any malfunction can result in immediate bodily or psychological harm (Gilbert, 2014). Thus, incorporation gives rise to shifting levels of transparency: on “good” days (Charmaz, 1991) the technology recedes into the background, while on “bad” days (Ιbid.) it becomes acutely present in the body. In this way, the benefits of DBS can simultaneously generate vulnerabilities (Goering et al., 2021), which may manifest as harms. Vulnerabilities often arise when maintenance practices are poorly executed or significantly delayed, failing to ensure adequate care. The technological components require regular adjustment to patients’ fluctuating needs, and the battery must be replaced periodically (Pateraki, 2022, 2023). These maintenance processes are integral to treatment. Consequently, lack of maintenance may result in iatrogenic harm that must be addressed. Several types of harms related to DBS maintenance were documented in the field. In the following sections, we elaborate a typology of these potential harms.

### Harms Related to Maintenance

With neurostimulation, specific practices related to maintenance can raise potential harms for its users. The first category of harms concerns calibration. DBS is not a one-step procedure; the device requires ongoing adjustment of stimulation parameters, typically performed every six months. Nikos, a 53-year-old man, has been living with DBS for almost three years. Despite proper device placement, he was unable to benefit because the stimulation parameters remained unadjusted for over a year.

Unfortunately, the calibration by the first doctor, the neurologist of the public hospital X, was a failure, because he did not perform a brain mapping, as my second one did. So, after a year and a half after the operation … I went to another doctor to see if he could calibrate me better, because I thought the potential of the device was not fully exploited.

(Interview with Nikos)

It took more than one year for Nikos to benefit from neurostimulation, meaning that an initial mis-calibration of the device resulted in harm, including a failure to alleviate symptoms. Instead, recurrent and rebound symptoms, along with psychological effects, emerged, aggravating his condition. As he explained during the interview: “Before my proper calibration, I was a mess! The symptoms were out of control! I was depressed and incapable of doing things. I was not functional at all! After my second doctor, I am very well.” (Interview with Nikos).

The case of Nikos illustrates why calibration should be considered a form of maintenance and a crucial practice through which the incorporation of DBS is continuously negotiated and sustained. The iterative practice of calibrating the DBS system constitutes what Young calls “a creative and transformative technical activity upon which the efficiency and reliability of technologies often depend” (Young, 2020, p. 364). Here, we see a first potential source of harm. Even when calibration is well performed, its experimental dimension can lead to adverse effects for the implanted patient. For example, Nikos experienced a severe outbreak of bipolar disorder during a calibration session (Pateraki, 2022, 2023). More broadly, this process can result in reversible and transient harms for patients.

A second type of harm relates to the IPG or battery replacement, showing how technological treatments depend on broader sociotechnical and economic contexts. The field study in Greece revealed that, in several cases, a seamless continuation of DBS treatment was impeded by financial constraints during the healthcare budgetary crisis of the 2010's (Pateraki, 2019, 2022, 2023, 2026).

Delayed IPG replacement can have devastating effects on the patients. Valia, a 73-year-old woman, had to wait 14 months for a replacement. Diagnosed with PD 23 years ago and undergoing DBS for the past 8 years, she has experienced all the stages of PD. At the time of the interview, she was bedridden with dementia and speech impediments. Speaking about her experience, her daughter and caregiver Thalia, described this prolonged wait as profoundly harmful, both physically and psychologically. During this period, many motor symptoms previously alleviated by DBS returned and worsened, and Valia developed dementia. Financial restrictions led to a lack of DBS maintenance and, consequently, severe incapacitation. This break in Valia’s treatment raises ethical concerns.

Existing literature on post-trial and post-implantation obligations emphasizes that the duties and responsibilities of researchers and trial organizers are central, ensuring the ongoing function of neuro-implantable devices and the prevention of avoidable harm to participants (e.g. Dasgupta et al., 2024; Lázaro-Muñoz et al., 2022; Fins et al., 2024). However, this literature does not specifically address maintenance issues in older devices within resource-limited healthcare systems. Valia’s case exemplifies how failing to uphold these obligations can be ethically problematic, showing that delays in replacement are not merely administrative matters but constitute serious ethical breaches that compromise standards of technological care.

As Antonia’s access to appropriate care was compromised, she recounts the harm she experienced waiting for her DBS to be maintained: “I stayed for almost a year with an idle DBS in my body, without battery. It was really tough. (…) Since they initially approve an implantation, they should also take care of the replacement of the batteries beforehand. It’s their duty!” (Interview with Antonia) The hospital administrator’s ethical stance on withholding treatments was rooted in budgetary considerations. She defended her decision, contending that the exorbitant cost of the IPG outweighed the necessity of Antonia’s DBS operation, as other priorities took precedence.

“The director of the hospital wouldn’t authorize the expense for the battery change. And she said: ‘she won’t die if her DBS has no battery, while a cancer patient will, if he doesn’t receive his expensive treatment.’ She won’t die, but she’ll be a mess … They should care for DBS, care for us since day one. Since they perform the implantation, they should keep supporting us, or not perform it at all!”.

(Interview with Antonia)

This vignette illustrates the ethical tension in healthcare decisions that deprioritize DBS maintenance. While prioritizing cancer patients over DBS users may be understandable in terms of maximizing the survival of the former, this does not diminish the harms experienced by the latter whose treatment is interrupted. Many patients and caregivers, including Thalia, report that such lapses worsen illness, increase vulnerability and compromise well-being. Healthcare providers must avoid causing harm. Discontinuation of essential DBS maintenance, leading to physical, psychological, and functional decline, constitutes a breach of this obligation and exposes patients to preventable risks.

A third type of harm related to maintenance involves faulty devices. These involve implants later recalled by the manufacturers. Orestis, a 55-year-old man who underwent surgery six years ago, received a device that had faulty leads. As he explained:

My implant was faulty and the manufactured company recalled it. … That happened seven months - I think it was seven- after my implantation. There was something wrong with the leads- if I remember well (…). The only thing I know for sure is that the method didn’t work! The stimulation didn’t work! My symptoms weren’t under control as I was expected. I was suffering from the disease and I was very frustrated. It was very stressful the idea I had to be operated again, once more time!

(Interview with Orestis)

Although Orestis was initially unable to benefit from neurostimulation due to a faulty device, this lack of therapeutic effect does not constitute direct harm but rather an indirect form of harm. While the device did not worsen his condition, it failed to restore functionality, leaving him vulnerable and dependent without relief. During the eight months until his second operation, when new leads were implanted and the device properly calibrated, he experienced intense psychological distress, illustrating the indirect harms associated with DBS. Even after the device was correctly adjusted, he continues to feel profound mistrust toward his implanted technology, highlighting the enduring ethical and emotional consequences of technological malfunction.

A fourth type of potential harms related to DBS involves surgery-linked infections. Themis, a 58-year-old, had a successful implantation, but the chest wound created for IPG placement became infected.

A problem emerged after the implantation of the battery. The wound was infected by bacteria. ‘Colonized bacteria’ as my treating neurologist used to say. I was treated with antibiotics. My doctor also disinfected my wound. Finally, it worked, but I was suffering! It was swelling and painful … I still remember the pain- and that awful smell.

(Interview with Themis)

In DBS, infections are always a possibility, as the invasive nature of the under-the-skin technology requires multiple incisions and precise surgical work, creating persistent vulnerabilities that demand careful management. Such imminent and foreseeable harms, common in clinical practice, are inherent to device placement. However, in chronic illnesses such as PD, there comes a point when treatment loses effectiveness, and continued maintenance may constitute overtreatment (Gilbert & Lancelot, 2021).

### Beyond Maintenance: DBS as Overtreatment and the Value of Caregivers

As discussed in section “Introduction”, maintenance in the case of DBS depends on successful incorporation and long-term durability. When PD reaches an advanced stage, continuing DBS treatment can become problematic, as harms may outweigh benefits. In patients in the final stages of PD, DBS loses its effectiveness and thus constitutes overtreatment, the continuation of a therapy that is no longer beneficial or desirable (Beauchamp & Childress, 2013, p. 160). One case is the aforementioned Valia, bedridden with speech impediments and dementia. As her daughter explained, neither DBS nor medication is effective anymore. Thalia recounted: “Unfortunately, the last year she is like a living-dead! … Nothing works anymore … She can’t move, she can’t swallow and eat, she can’t go to the toilet, she doesn’t understand or communicate … That’s why I call her a living dead!” (Interview with Thalia)

Another example is John, one of the earliest patients in Greece to undergo DBS. Diagnosed at 37 with Young Onset Parkinson’s Disease (YOPD), he lived with his caregiver Lina, a migrant who had care for him professionally since the start of his DBS treatment. As with other patients, John’s speech difficulties posed significant challenges during the interview, with his answers usually limited to “yes” or “no.” To facilitate communication and ensure engagement, the interviewer developed an easy-language questionnaire, enabling patients like John to respond despite their verbal limitations.

Lina often spoke on John’s behalf, although they frequently disagreed silently through subtle non-verbal cues such as eye movements, glances, facial expressions, and shifts in posture. These interactions highlight “relational agency,” in which agency is understood as distributed across the patient and caregiver rather than residing solely in the individual (Klein & Goering, 2023). When Lina spoke for John, moments of relational agency emerged, while John’s non-verbal signals revealed nuances of disagreement or discomfort that verbal responses alone could not capture. Following the dyadic analysis proposed by Boulicault et al. (2023), responses we evaluated by considering verbal and non-verbal elements, as well as individual experiences and interactions. In the following instance, John’s silent disagreements and expressions revealed the intense embarrassment and discomfort he felt in response to Lina’s words.

Lina: Sometimes, especially in the beginning, I used to tamper with the controller. I used to change minor parameters of the stimulation. Not anymore, not that often, because the results are not visible.

Interviewer: Can you explain to me what do you mean?

Lina: Yes! Well, as his PD progressed and other symptoms came to the fore, playing with the stimulation didn’t help him anymore … I mean, in the last years his speech has deteriorated, and he has cognitive problems that stimulation can’t ease. In this stage, as a caregiver, I offer him more than this device! Maybe future devices will be better than caregivers, but now they aren’t!

(Interview with Lina)

Lina, accustomed with the technology, is frustrated by its declining effectiveness. As DBS increasingly fails to improve John’s condition, her caregiving work becomes even more burdensome. She further explains:

You see him! He has dementia. The neurostimulation doesn’t work anymore, as it did at the first years. Medication doesn’t help! He is like a stubborn child! He stucks, he doesn’t understand anymore for example easy tasks I’m explaining him. He has lost completely the sense of time. He can’t decide about easy tasks, for example to choose between two meals for me to cook! But it’s worst when he is walking, for it is impossible for him to balance himself! He has been hospitalized twice after falling, and I have to follow him in his every step, in order to keep him alive.

(Interview with Lina)

Thus, even with regular and effective maintenance, DBS has limits. According to Eran Klein and Sara Goering (2023), who focus on dementia, caregiver burden is multidimensional, encompassing daily supervision, decision-making, preventive safety measures, and psychological pressure from social and cultural expectations. Effectively recognizing and distributing this burden through supportive systems is essential to protect both caregivers and the individuals they care for. Continuing an ineffective treatment only adds to patients’ and caregivers’ burdens, marking a clear case of overtreatment. As pointed out in studies, even DBS’s success generates ethical problems: longevity gains are offset by new stages of new stages of PD and the emergence of “iatrogenic” harms. It is suggested that “many symptoms of PD are reaching more severe stages that are lasting longer” (Gilbert & Lancelot, 2021, p. 1). This generation of iatrogenic harms introduces ethical challenges that go beyond the one of proper maintenance of the body fitted with DBS.

### Ηarms Related to Maintenance

So far, several types of harms related to DBS maintenance have emerged. A short section listing and categorizing these harms is provided in section “Patients’ Narratives: Evidence of How Harm Intertwines with Maintenance” (and its subsections), including some not directly captured in interviews but are nonetheless significant. As described above, they are classified in Table 1, which consolidates harms identified through the empirical study in Greece and those documented in secondary literature.

**Table 1:** Types of harms related to maintenance

Type of Harm	Description	Reversibility	Duration
A. Calibration-related harms	Issues during calibration sessions	Reversible	Transient
B. Miscalibration harms	Incorrect stimulation settings	Reversible	Transient
C. Delayed IPG/battery replacement	Interruptions in therapy due to delays	Reversible or Irreversible	Transient or Chronic
D. Faulty implanted components	Defective IPG, leads, electrodes, burr-hole caps	Reversible	Transient
E. EMI exposure harms	Adverse effects from electromagnetic interference	Reversible	Transient
F. Severe surgical complications	Infections, perforation, leakage, hemorrhage, migration	Reversible or Irreversible	Transient or Chronic
G. Overtreatment harms	Continuation of ineffective DBS in late-stage PD	Irreversible	Chronic

DBS treatment can give rise to a variety of harms across different circumstances. For instance, miscalibration of the device can lead to a broad range of adverse outcomes (Pateraki, 2022, 2023). These include harms involving physical and psychological effects, often reversible, as well as harms caused by miscalibration that affect body and mind. Delays in IPG or battery replacement can produce both reversible and irreversible physical and psychological symptoms (Section “Harms Related to Maintenance”, Pateraki, 2022, 2023). Faulty technological components of DBS, such as IPGs, leads, electrodes, or burr-hole caps, can also induce harms, typically reversible. Patients may additionally experience adverse effects arising from exposure to electromagnetic interference (EMI) environments, with generally reversible physical and psychological consequences (Goering et al., 2021; Pateraki, 2019, 2022, 2023; Rahimpour et al., 2021). More severe harms may occur due to infections, subcutaneous perforation, implant leakage, hemorrhage, or implant migration, potentially resulting in chronic or transient, reversible or irreversible symptoms (Section “Harms Related to Maintenance”, Maslen et al., 2018, Pateraki, 2019, 2022). In catastrophic cases, inadequate immediate response to such harms can escalate to tragic outcomes, including suicide attempts or completions, as noted in reports by coroners and further research (Coroners Court of Queensland, 2017; State Coroner’s Court of New South Wales, 2014; Gilbert, 2013). Finally, overtreatment can lead to chronic, irreversible physical and/or psychological harms. This highlights the critical need for timely and effective management of potential iatrogenic harms to mitigate their impact on patients.

Collectively, these categories illustrate the complex spectrum of iatrogenic risks associated with DBS and emphasize the importance of care requirements. Iatrogenic harms, or adverse effects resulting from medical interventions, as illustrated in Table 1, can manifest in a range of severities and dimensions. These effects may be transient or chronic and can vary in intensity from mild to severe. The spectrum of iatrogenic harms thus encompasses physical, psychological, personal, and social consequences, and extends beyond what this article suggests.

## Conclusion

This study expands the scope of neuroethical inquiry by addressing iatrogenic harms related to DBS maintenance, highlighting the significance of ongoing care. By examining bodies with neural technology as infrastructures requiring constant attention, we show that harms can emerge not only from device malfunction but also from inadequate, delayed, or poorly executed maintenance. Drawing on patient narratives and secondary literature, we developed a typology of harms, including miscalibration, device failures, infections, and overtreatment, illustrating the vulnerabilities patients face and the trade-offs inherent in living with invasive neurotechnologies.

Focusing on older DBS devices in a resource-constrained healthcare system reveals a gap in current research, which often prioritizes innovation over long-term maintenance. Our findings demonstrate that inadequate maintenance can lead to recurrent and rebound symptoms, along with psychological effects, imposing unforeseen burdens on both patients and caregivers (Gilbert et al., 2024). These results emphasize the ethical responsibilities of healthcare providers in supporting technological bodies in chronic illnesses.

Finally, this study highlights the inherent limitations of technological interventions in managing chronic illness and the enduring value of caregiving. It calls for a deeper appreciation of the ethical dimensions associated with the maintenance of neural implants and the broader implications for patient care, ensuring therapeutic benefits without compromising patient well-being.

## Data Availability

Not applicable.
